# Supplementary material for: In depth transcriptomic profiling defines a landscape of dysfunctional immune responses in patients with VEXAS syndrome
Source: Nat Commun. 2025 May 20;16:4690. doi: 10.1038/s41467-025-59890-0 (PMC12092610; doi:10.1038/s41467-025-59890-0)
Supplement: Supplementary file 1 — Supplementary Information [file 41467_2025_59890_MOESM1_ESM.pdf]

SUPPLEMENTARY INFORMATION

In depth transcriptomic profiling defines a landscape of dysfunctional immune responses in patients with VEXAS syndrome

Hiroki Mizumaki,[1,*] Shouguo Gao,[1,*] Zhijie Wu,[1] Fernanda Gutierrez-Rodrigues,[1] Massimiliano Bissa,[2] Xingmin Feng,[1] Emma M. Groarke,[1] Haoran Li,[1] Lemlem Alemu,[1] Diego Quinones Raffo,[1] Ivana Darden,[1] Sachiko Kajigaya,[1] Peter C. Grayson,[3] Genoveffa Franchini,[2] Neal S. Young,[1,†] and Bhavisha A. Patel,[1,†]

[*] These authors contributed equally.

[†] These authors jointly supervised this work.

[1] Hematology Branch, National Heart, Lung, and Blood Institute, National Institutes of Health, Bethesda, MD, United States

[2] Animal Models and Retroviral Vaccines Section, National Cancer Institute, National Institutes of Health, Bethesda, MD, United States

[3] Vasculitis Translational Research Program, National Institute of Arthritis and Musculoskeletal, and Skin Diseases, National Institutes of Health, Bethesda, MD, United States

Supplementary Tables

Supplementary Table 1. Clinical and laboratory characteristics of patients with VEXAS syndrome

Supplementary Table 2. *UBA1* genotyping metrics in 10x gene expression data and GoT libraries

Supplementary Table 3. Differentially expressed genes in monocytes from VEXAS

Supplementary Table 4. Differentially expressed genes in *UBA1*-mutated NK cells

Supplementary Table 5. Sequences of TCR groups with more than five different clones obtained from GLIPH2 analysis

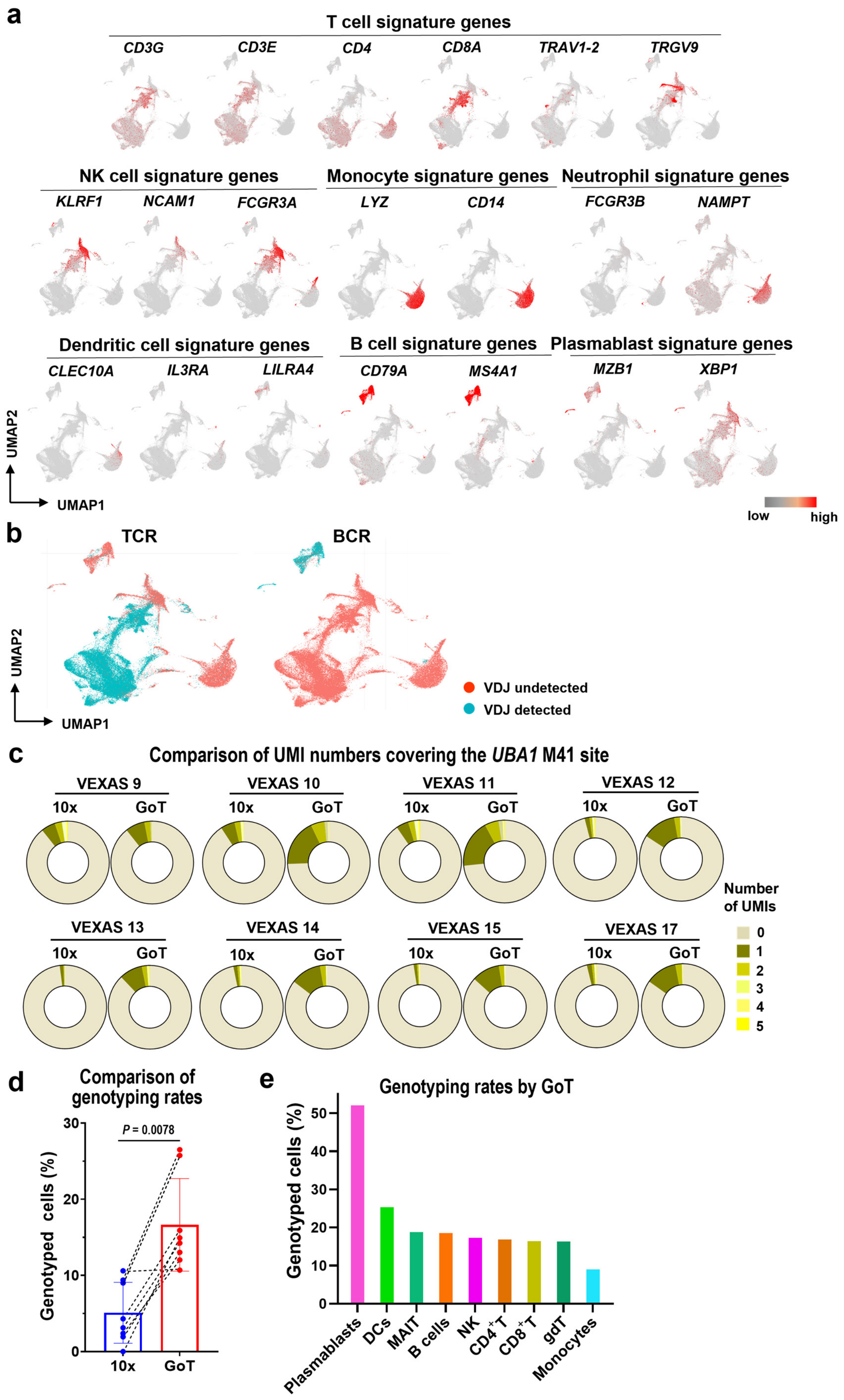

a
T cell signature genes
CD3G
CD3E
CD4
CD8A
TRAV1-2
TRGV9
NK cell signature genes
KLRF1
NCAM1
FCGR3A
Monocyte signature genes
LYZ
CD14
Neutrophil signature genes
FCGR3B
NAMPT
Dendritic cell signature genes
CLEC10A
IL3RA
LILRA4
B cell signature genes
CD79A
MS4A1
Plasmablast signature genes
MZB1
XBP1
UMAP2
UMAP1
low
high
b
TCR
BCR
UMAP2
UMAP1
VDJ undetected
VDJ detected
c
Comparison of UMI numbers covering the UBA1 M41 site
VEXAS 9
VEXAS 10
VEXAS 11
VEXAS 12
VEXAS 13
VEXAS 14
VEXAS 15
VEXAS 17
10x
GoT
Number of UMIs
0
1
2
3
4
5
d
Comparison of genotyping rates
P = 0.0078
Genotyped cells (%)
0
10
20
30
10x
GoT
e
Genotyping rates by GoT
Genotyped cells (%)
0
10
20
30
40
50
Plasmablasts
DCs
MAIT
B cells
NK
CD4+T
CD8+T
gdT
Monocytes

Supplementary Fig. 1: Cell type annotation and genotyping of PBMCs from VEXAS patients. **a**, Expression of cell-type specific genes highlighted in uniform manifold approximation and projection (UMAP) plots of peripheral blood mononuclear cells (PBMC) from all VEXAS patients and healthy donors. **b**, Expression of TCR (left) and BCR (right) highlighted in UMAP plots of PBMCs of all VEXAS patients and healthy donors. **c**, Fractions of cells by the numbers of unique molecular identifier (UMI) with the *UBA1 M41* locus coverages in standard 10x gene expression data processed with cb_sniffer (left) and UMIs with *UBA1 M41* locus coverages in genotyping of transcriptomes (GoT) amplicon libraries (right) (see Supplementary Table 2 for cell numbers in each sample). **d**, Comparison of genotyping rates obtained from standard 10x gene expression data (n = 8) and GoT data (n = 8). Data are presented as a mean value with SD. *P* values were calculated using the two-sided Wilcoxon matched-pairs signed rank test. **e** Genotyping rates through GoT in major cell types. Cell types with more than 100 cells were analyzed. DCs, dendritic cells; MAIT, mucosa-associated invariant T cell; gdT, gamma delta T cells.

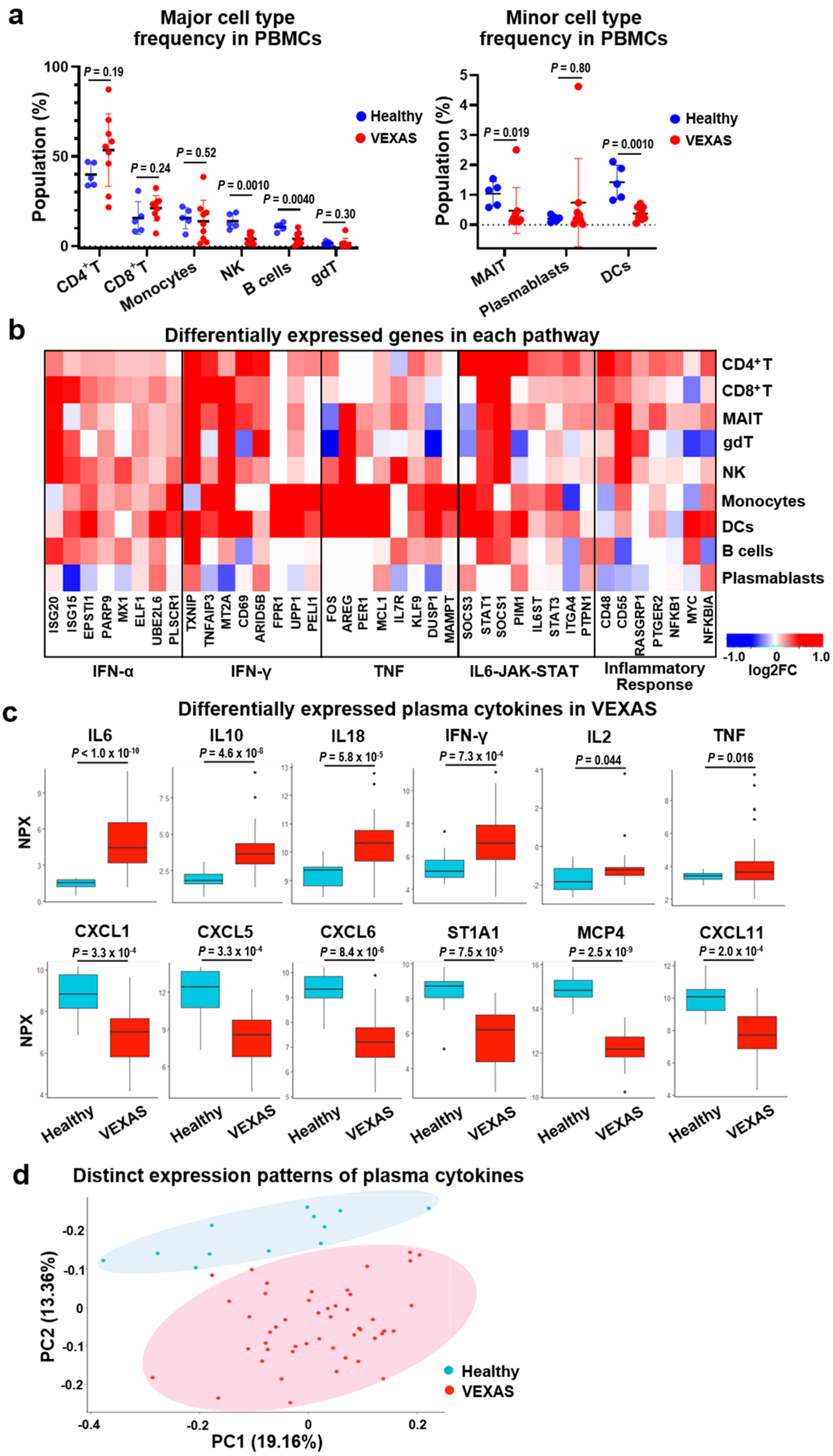
a
Major cell type
frequency in PBMCs
Population (%)
P = 0.19
P = 0.24
P = 0.52
P = 0.0010
P = 0.0040
P = 0.30
CD4+T
CD8+T
Monocytes
NK
B cells
gdT
Healthy
VEXAS
Minor cell type
frequency in PBMCs
P = 0.019
P = 0.80
P = 0.0010
MAIT
Plasmablasts
DCs
b
Differentially expressed genes in each pathway
CD4+T
CD8+T
MAIT
gdT
NK
Monocytes
DCs
B cells
Plasmablasts
ISG20
ISG15
EPSTI1
PARP9
MX1
ELF1
UBE2L6
PLSCR1
TXNIP
TNFAIP3
MT2A
CD69
ARID5B
FPR1
UPP1
PELI1
FOS
AREG
PER1
MCL1
IL7R
KLF9
DUSP1
MAMPT
SOCS3
STAT1
SOCS1
PIM1
IL6ST
STAT3
ITGA4
PTPN1
CD48
CD55
RASGRP1
PTGER2
NFKB1
MYC
NFKBIA
IFN-α
IFN-γ
TNF
IL6-JAK-STAT
Inflammatory
Response
-1.0
0
1.0
log2FC
c
Differentially expressed plasma cytokines in VEXAS
IL6
P < 1.0 x 10-10
IL10
P = 4.6 x 10-8
IL18
P = 5.8 x 10-5
IFN-γ
P = 7.3 x 10-4
IL2
P = 0.044
TNF
P = 0.016
NPX
CXCL1
P = 3.3 x 10-4
CXCL5
P = 3.3 x 10-4
CXCL6
P = 8.4 x 10-6
ST1A1
P = 7.5 x 10-5
MCP4
P = 2.5 x 10-9
CXCL11
P = 2.0 x 10-4
Healthy
VEXAS
d
Distinct expression patterns of plasma cytokines
PC2 (13.36%)
PC1 (19.16%)
Healthy
VEXAS

Supplementary Fig. 2: Differences in cellular composition, gene expression, and cytokine production between VEXAS patients and healthy donors. **a**, Percentages of each major cell type relative to the total number of PBMCs derived from VEXAS patients (n = 9, red dots) and healthy donors (n = 5, blue dots): major cell types with an average ratio of 2% or greater (left), and less than 2% (right). Data are presented as a mean value with SD. *P* values were calculated using the two-sided unpaired Mann-Whitney *U* test. **b**, Heatmap showing expression of differentially expressed representative genes grouped by their functional pathways in IFN-α signaling, IFN-γ signaling, TNF signaling, IL6-JAK-STAT signaling, and inflammatory response pathways among the major cell types of VEXAS patients (n = 9) as compared to those of healthy donors (n = 5). Values are presented as log2-fold change (FC). **c**, Box and whisker plots showing normalized plasma protein expression (NPX) levels of IL-6, IL-10, IL-18, IFN-γ, IL-2, TNF, CXCL1, CXCL5, CXCL6, ST1A1, MCP4, and CXCL11. A box plot shows median and interquartile ranges (IQR); Lower and upper hinges correspond to the first and third quartiles. A upper whisker extends from the hinge to the largest value no further than 1.5*IQR from a hinge. A lower whisker extends from the hinge to the smallest value at most 1.5*IQR from the hinge. *P* values were calculated using the two-sided unpaired Mann-Whitney *U* test. **d**, Principal component (PC) analysis plot of individual plasma samples based on cytokine expression levels measured by the Olink inflammatory panel multiplex immunoassay. Each dot represents one individual sample from VEXAS patients (red dots) and from healthy donors (blue dots). DCs, dendritic cells; MAIT, mucosa-associated invariant T cells; gdT, gamma delta T cells.

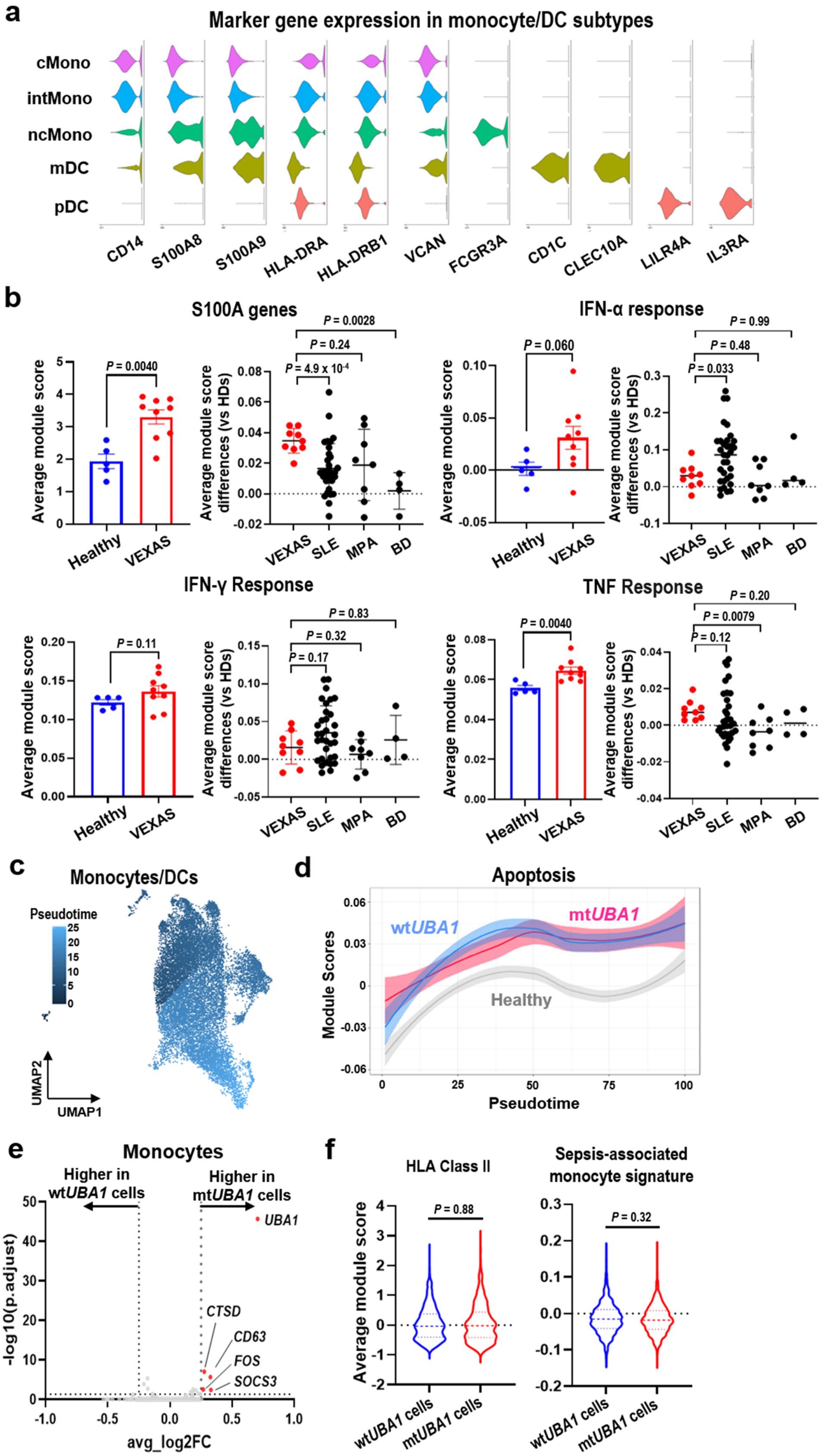

a
Marker gene expression in monocyte/DC subtypes
cMono
intMono
ncMono
mDC
pDC
CD14
S100A8
S100A9
HLA-DRA
HLA-DRB1
VCAN
FCGR3A
CD1C
CLEC10A
LILR4A
IL3RA
b
S100A genes
IFN-α response
IFN-γ Response
TNF Response
Average module score
Average module score differences (vs HDs)
Healthy
VEXAS
SLE
MPA
BD
P = 0.0040
P = 0.0028
P = 0.24
P = 4.9 x 10-4
P = 0.060
P = 0.99
P = 0.48
P = 0.033
P = 0.11
P = 0.83
P = 0.32
P = 0.17
P = 0.0040
P = 0.20
P = 0.0079
P = 0.12
c
Monocytes/DCs
Pseudotime
UMAP2
UMAP1
d
Apoptosis
wtUBA1
mtUBA1
Healthy
Module Scores
Pseudotime
e
Monocytes
Higher in wtUBA1 cells
Higher in mtUBA1 cells
UBA1
CTSD
CD63
FOS
SOCS3
-log10(p.adjust)
avg_log2FC
f
HLA Class II
Sepsis-associated monocyte signature
P = 0.88
P = 0.32
Average module score
wtUBA1 cells
mtUBA1 cells

Supplementary Fig. 3: Immunological features of monocytes in VEXAS. a, Violin plot showing expression distributions of selected canonical markers in five subtypes of monocytes and dendritic cells (DC). Rows and columns represent selected marker genes and cell types, respectively. **b**, Gene module scores of alarmin-related S100A genes, IFN-α response, IFN-γ response, and TNF response pathways in monocytes across VEXAS (n = 9), healthy donors (n = 5), and other multisystem autoimmune diseases (Systemic lupus erythematosus (SLE), n = 33; microscopic polyangiitis (MPA), n = 8; Behcet's disease (BD), n = 4). For comparison among diseases, differences of gene module scores between VEXAS and healthy donors are displayed. Data are presented as mean with SD. *P* values were calculated using two-sided unpaired Mann-Whitney *U* test. **c**, UMAP plot visualizing pseudotime trajectories of all monocytes/DCs in VEXAS. A color code indicates cellular moments in the calculated pseudotime. **d,** Dynamic changes of gene module scores of the apoptosis pathway in wild-type *UBA1* (wt*UBA1*) and *UBA1*-mutated (mt*UBA1*) monocytes from VEXAS and monocytes from healthy donors along differentiation. Data are presented as a mean value with 1.96*SE. x axis, pseudotime ordering from classical monocytes to nonclassical monocytes estimated by slingshot; y axis, gene module scores of apoptosis pathway. **e**, Volcano plots of differentially expressed genes between wt*UBA1* and mt*UBA1* monocytes. A horizontal dotted line and vertical dotted lines represent an adjusted *p* value (*p*adj) = 0.05 and absolute log2FC = 0.25, respectively. Genes upregulated in mt*UBA1* monocytes (red) are highlighted. *P* values were calculated with a two-sided Wilcoxon rank-sum test and Bonferroni correction for multiple comparisons. **f,** Gene module scores in wt*UBA1*and mt*UBA1* monocytes for HLA class II genes and sepsis-associated monocyte signature scores. *P* values were calculated using the two-sided unpaired Mann-Whitney *U* test.

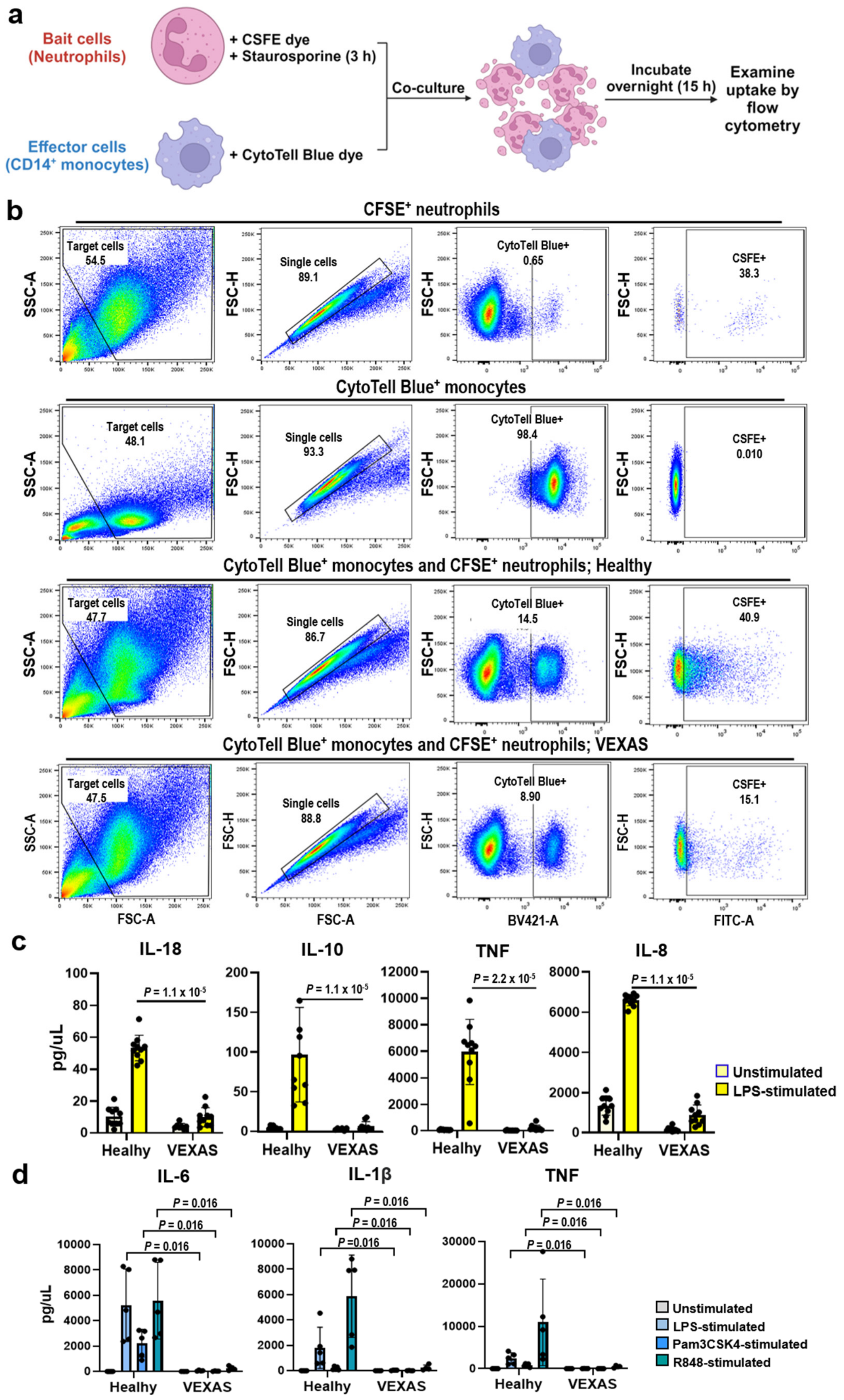
a
Bait cells (Neutrophils)
+ CSFE dye
+ Staurosporine (3 h)
Co-culture
Incubate overnight (15 h)
Examine uptake by flow cytometry
Effector cells (CD14+ monocytes)
+ CytoTell Blue dye
b
CFSE+ neutrophils
Target cells 54.5
Single cells 89.1
CytoTell Blue+ 0.65
CSFE+ 38.3
CytoTell Blue+ monocytes
Target cells 48.1
Single cells 93.3
CytoTell Blue+ 98.4
CSFE+ 0.010
CytoTell Blue+ monocytes and CFSE+ neutrophils; Healthy
Target cells 47.7
Single cells 86.7
CytoTell Blue+ 14.5
CSFE+ 40.9
CytoTell Blue+ monocytes and CFSE+ neutrophils; VEXAS
Target cells 47.5
Single cells 88.8
CytoTell Blue+ 8.90
CSFE+ 15.1
SSC-A
FSC-H
FSC-A
BV421-A
FITC-A
c
IL-18
IL-10
TNF
IL-8
pg/uL
P = 1.1 x 10-5
P = 1.1 x 10-5
P = 2.2 x 10-5
P = 1.1 x 10-5
Healhy
VEXAS
Unstimulated
LPS-stimulated
d
IL-6
IL-1β
TNF
pg/uL
P = 0.016
Healhy
VEXAS
Unstimulated
LPS-stimulated
Pam3CSK4-stimulated
R848-stimulated

Supplementary Fig. 4: Functional in vitro assay using CD14$^+$ monocytes from VEXAS patients. **a**, Schematic representation of the efferocytosis assay. Neutrophils isolated from fresh blood of an unrelated healthy donor were stained with CSFE and induced apoptosis with Staurosporine. CD14$^+$ monocytes isolated from frozen peripheral blood mononuclear cells (PBMC) of VEXAS patients and healthy donors were stained with CytoTell Blue. CSFE-labeled apoptotic "bait" neutrophils were then cocultured with "effector" CD14$^+$ monocytes overnight. Frequency of CSFE$^+$ apoptotic cell uptake in CytoTell Blue$^+$ efferocytes (CD14$^+$ monocytes) were measured with a flow cytometer. Figure was created in BioRender. Mizumaki, H. (2025) [https://BioRender.com/w79k163]. **b**, Representative flow plots showing a gating strategy for identifying efferocytes that engulfed apoptotic neutrophils. Cells were gated on monocytes/neutrophils, singlets, CytoTell Blue$^+$ and CFSE$^+$. Frequency of efferocytes was expressed as frequency of CFSE$^+$ cells in CytoTell Blue$^+$ monocytes. CFSE$^+$ neutrophils (upper panel) and CytoTell Blue$^+$ monocytes (second panel) not cocultured were used as controls for setting the gates. Representative third and bottom panels show cocultured CFSE$^+$ neutrophils and CytoTell Blue$^+$ monocytes from a healthy donor and a VEXAS patient, respectively. **c**, Cytokine detection of IL-18, IL-10, TNF, and IL-8 in culture supernatants of purified CD14$^+$ monocytes (VEXAS patients, n = 10; healthy donors, n = 10) after 8 h in-vitro incubation with or without 100 ng/mL of lipopolysaccharide (LPS). Data are presented as a mean value with SD. *P* values were calculated using the two-sided unpaired Mann-Whitney *U* test. **d**, Cytokine detection of IL-6, IL-1β, and TNF in culture supernatants of purified CD14$^+$ monocytes (VEXAS patients, n = 4; healthy donors, n = 5) after 8 h in-vitro incubation with or without 100 ng/mL of lipopolysaccharide (LPS), 100 ng/mL of Pam3CSK4, and 2.5 μM of R848. Data are presented as mean with SD. *P* values were calculated using the two-sided unpaired Mann-Whitney *U* test.

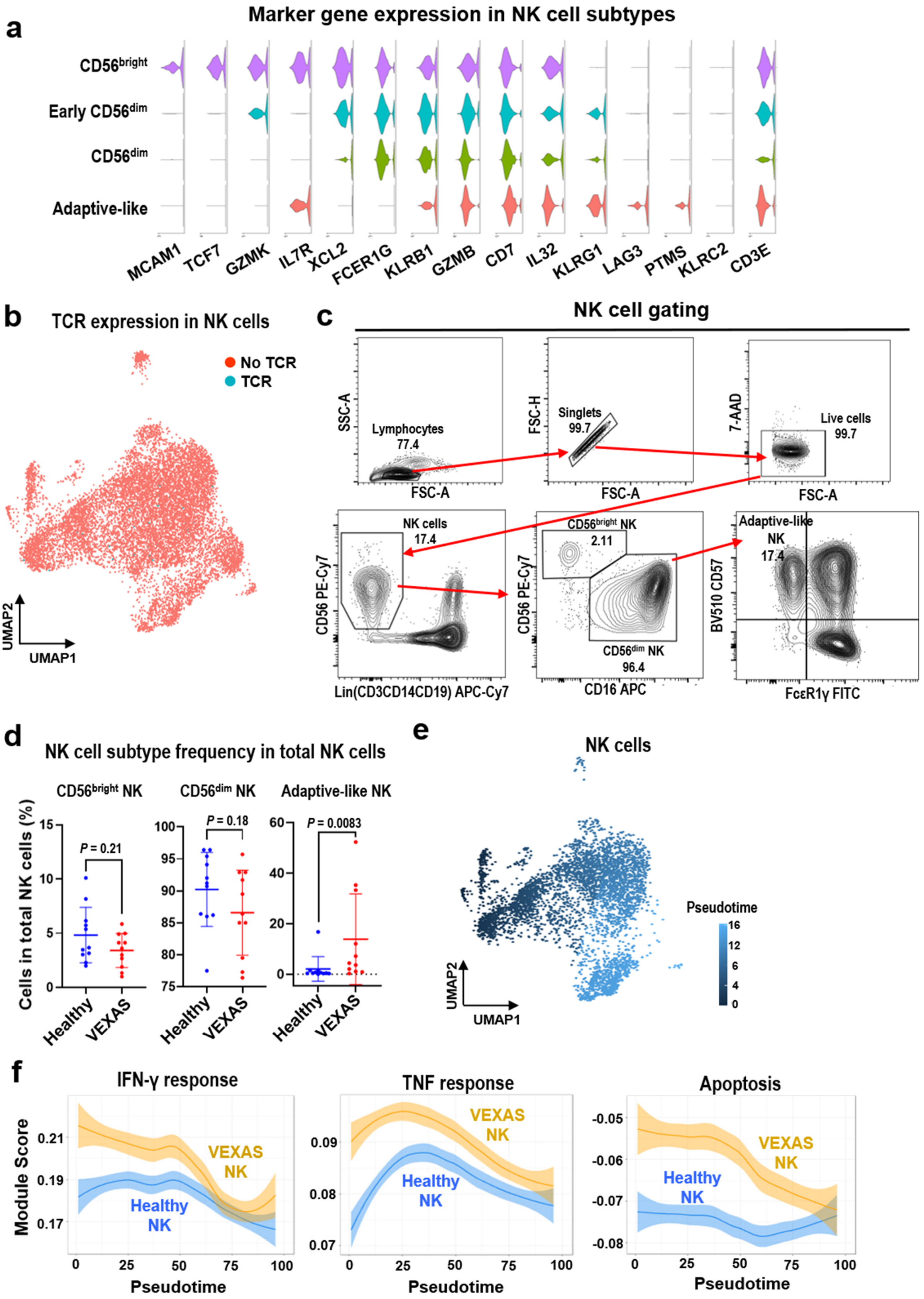
a
Marker gene expression in NK cell subtypes
CD56bright
Early CD56dim
CD56dim
Adaptive-like
MCAM1 TCF7 GZMK IL7R XCL2 FCER1G KLRB1 GZMB CD7 IL32 KLRG1 LAG3 PTMS KLRC2 CD3E
b
TCR expression in NK cells
No TCR
TCR
UMAP2
UMAP1
c
NK cell gating
SSC-A
Lymphocytes 77.4
FSC-A
FSC-H
Singlets 99.7
FSC-A
7-AAD
Live cells 99.7
FSC-A
NK cells 17.4
CD56 PE-Cy7
Lin(CD3CD14CD19) APC-Cy7
CD56bright NK 2.11
CD56dim NK 96.4
CD56 PE-Cy7
CD16 APC
Adaptive-like NK 17.4
BV510 CD57
FcεR1γ FITC
d
NK cell subtype frequency in total NK cells
CD56bright NK
CD56dim NK
Adaptive-like NK
Cells in total NK cells (%)
P = 0.21
P = 0.18
P = 0.0083
Healthy
VEXAS
e
NK cells
Pseudotime
UMAP2
UMAP1
f
IFN-γ response
TNF response
Apoptosis
Module Score
VEXAS NK
Healthy NK
Pseudotime

Supplementary Fig. 5: Immunological features of NK cells in VEXAS. a, Violin plot of gene expression distributions of selected canonical markers in the four NK cell subtypes. Rows and columns show selected marker genes and represent cell types. **b**, UMAP plot embedding of expression of TCR in subclustered NK cells. **c**, Representative flowcharts for identification of adaptive-like NK cells in peripheral blood. Adaptive-like NK cells were identified as (lowSSC/Singlets/Live/$CD56^{+}CD3^{-}CD14^{-}CD19^{-}$/$CD16^{+}CD56^{dim}$/$CD57^{+}Fc\varepsilon R1\gamma^{-}$). **d**, Proportions of NK cell subtypes relative to the total number of NK cells in healthy donors (n = 11, blue dots) and VEXAS patients (n = 11, red dots) by flow cytometry. Data are presented as a mean value with SD. *P* values were calculated using the two-sided unpaired Mann-Whitney *U* test. **e**, UMAP plot visualizing the pseudotime trajectory of all NK cells in VEXAS. Color code indicates cellular moments in the calculated pseudotime. **f**, Dynamic changes of gene module scores of the IFN-γ response, TNF#response and apoptosis pathways in NK cells from VEXAS patients and healthy donors along differentiation. Data are presented as a mean value with 1.96*SE. x axis, pseudotime ordering from $CD56^{bright}$ NK cells to adaptive-like NK cells estimated by slingshot; y axis, gene module scores for each pathway.

a

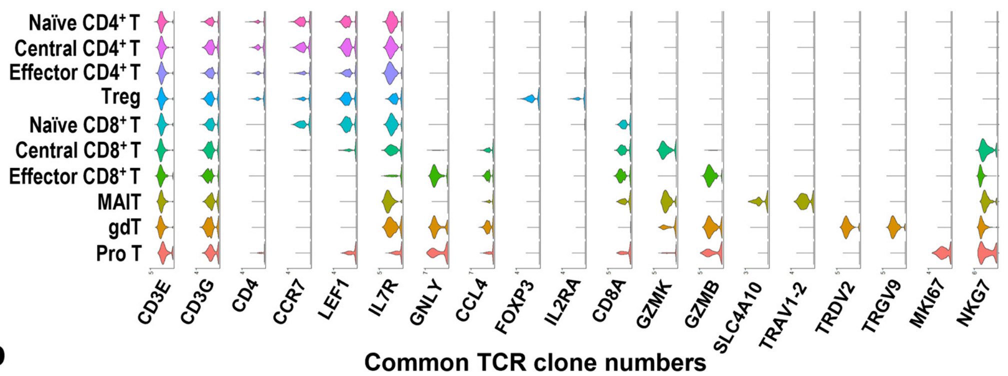

b

Common TCR clone numbers

		Healthy donors					VEXAS								
		#1	#2	#3	#4	#7	#9	#10	#11	#12	#13	#14	#15	#16	#17
Healthy donors	#1	200	5	7	5	5	4	2	3	1	0	1	0	2	6
	#2	5	200	5	2	5	3	3	2	1	0	1	2	2	5
	#3	7	5	200	5	5	4	4	4	1	0	1	1	2	8
	#4	5	2	5	200	2	4	3	3	2	0	0	1	3	3
	#7	5	5	5	2	200	4	5	6	2	0	1	1	2	6
VEXAS	#9	4	3	4	4	4	200	3	4	1	1	3	1	3	3
	#10	2	3	4	3	5	3	200	6	2	0	3	2	4	2
	#11	3	2	4	3	6	4	6	200	3	0	0	1	3	2
	#12	1	1	1	2	2	1	2	3	200	0	0	1	2	1
	#13	0	0	0	0	0	1	0	0	0	200	0	0	0	1
	#14	1	1	1	0	1	3	3	0	0	0	200	0	1	1
	#15	0	2	1	1	1	1	2	1	1	0	0	200	0	1
	#16	2	2	2	3	2	3	4	3	2	0	1	0	200	2
	#17	6	5	8	3	6	3	2	2	1	1	1	1	2	200

Number of shared TCR sequences: <10 | 10 - 50 | 50 - 100 | >100

c **d**

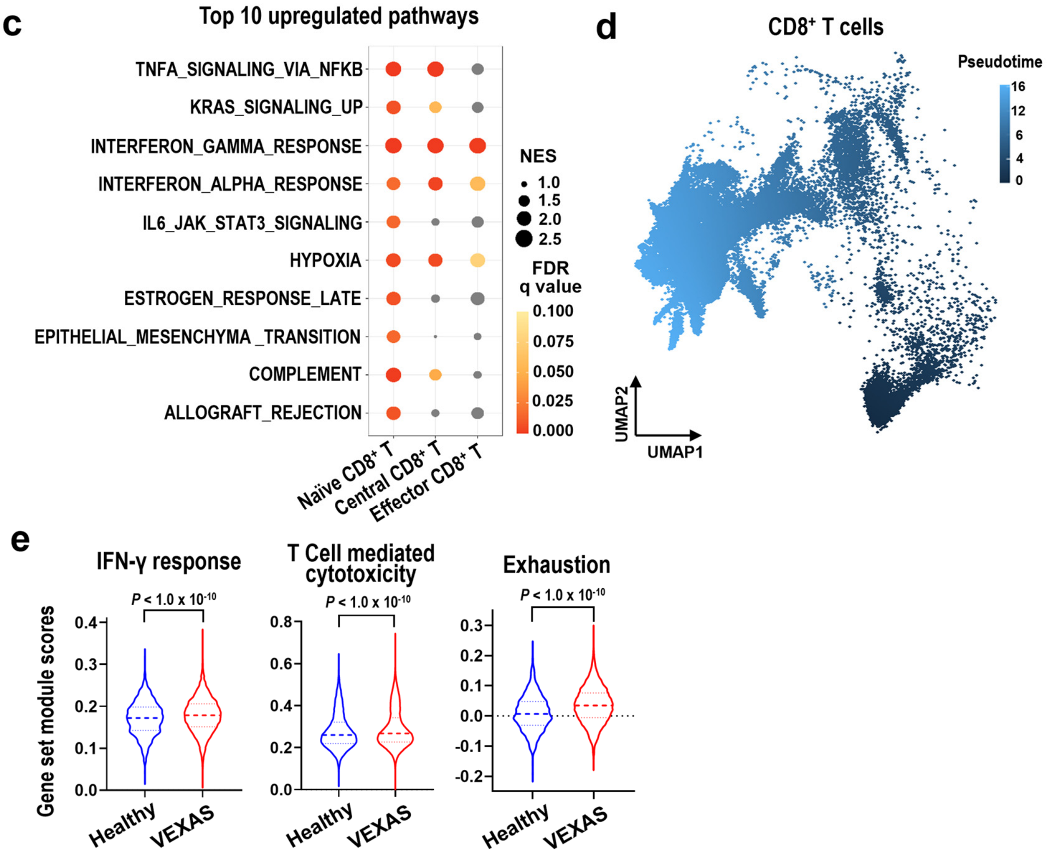

Supplementary Fig. 6: Immunological features of T cells and TCR analysis. a, Violin plot showing expression distributions of selected canonical cell markers in ten T cell subtypes. Rows and columns represent clusters and selected marker genes, respectively. **b**, Heatmap showing the numbers of common TCR clones in VEXAS patients (n = 9) and healthy donors (n = 5) among top 200 TCR clones. Both x- and y-axes indicate samples of VEXAS patients and healthy donors. Numbers indicate counts of identical TCR clones shared among samples. A color scheme ranging from dark orange to blue represents the numbers of shared CDR sequences from high to low. In general, there was few common TCR usage in VEXAS patients or healthy donors. There was also few common TCR usage among VEXAS patients and healthy donors. **c**, Dot plot showing gene set enrichment scores of top 10 upregulated hallmark pathways across $CD8^+$ T cell subtypes. Dot size indicates mean normalized enrichment score (NES) difference between VEXAS patients and healthy donors, and color scale indicates false discovery rate (FDR) value. Non-significant pathways (FDR $\geq$ 0.10) are in grey. **d**, UMAP plot illustrating the pseudotime trajectory of all $CD8^+$ T cells. A color code indicates cellular moments in the calculated pseudotime. **e**, Gene module scores in expanded $CD8^+$ T cells from healthy donors versus those of VEXAS patients for IFN-γ response, and T-cell mediated cytotoxicity and exhaustion pathways.

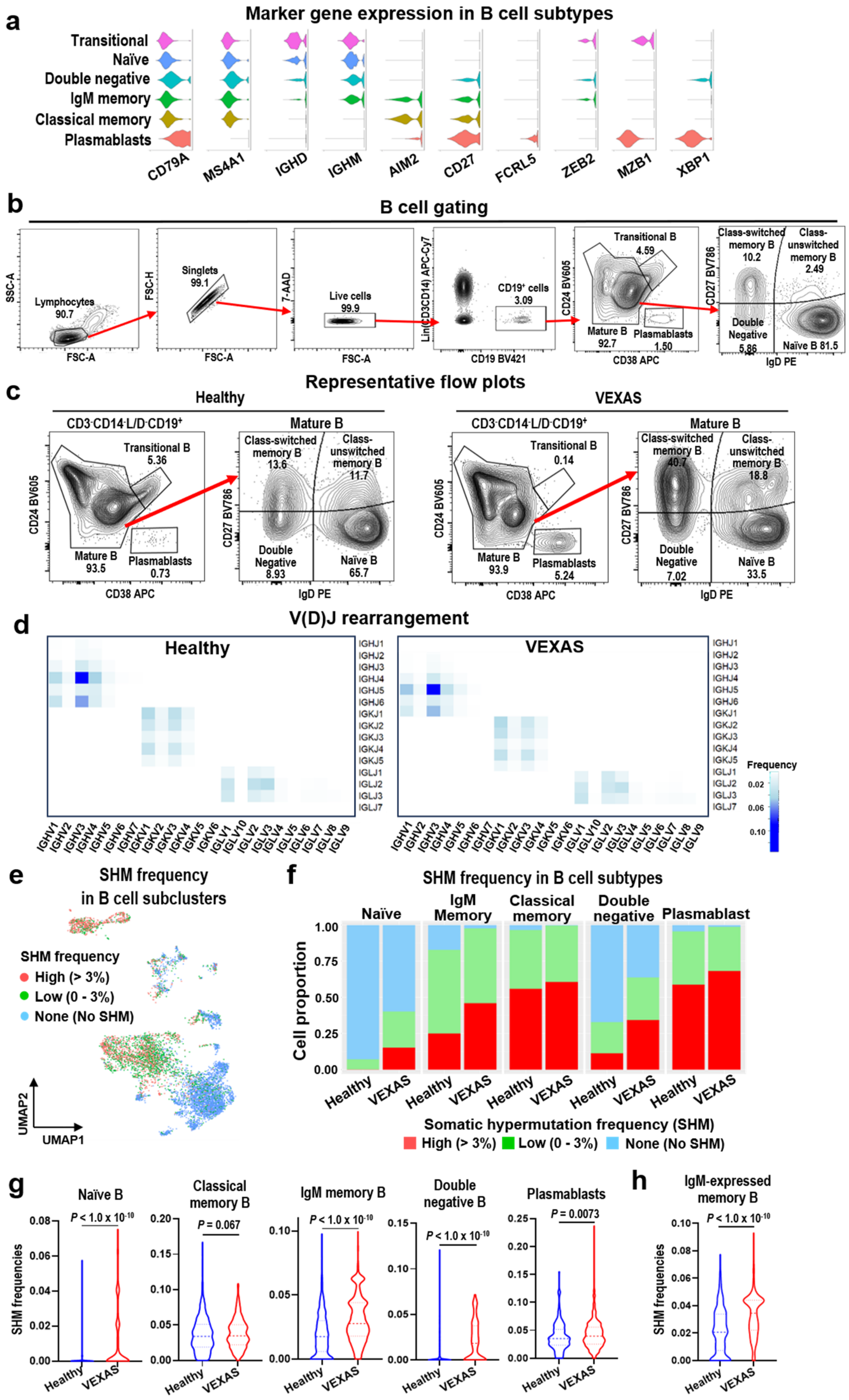
a
Marker gene expression in B cell subtypes
Transitional
Naïve
Double negative
IgM memory
Classical memory
Plasmablasts
CD79A
MS4A1
IGHD
IGHM
AIM2
CD27
FCRL5
ZEB2
MZB1
XBP1
b
B cell gating
Lymphocytes 90.7
Singlets 99.1
Live cells 99.9
CD19+ cells 3.09
Transitional B 4.59
Mature B 92.7
Plasmablasts 1.50
Class-switched memory B 10.2
Class-unswitched memory B 2.49
Double Negative 5.86
Naïve B 81.5
SSC-A
FSC-A
FSC-H
7-AAD
Lin(CD3CD14) APC-Cy7
CD19 BV421
CD24 BV605
CD38 APC
CD27 BV786
IgD PE
c
Representative flow plots
Healthy
VEXAS
CD3-CD14-L/D-CD19+
Mature B
Transitional B 5.36
Mature B 93.5
Plasmablasts 0.73
Class-switched memory B 13.6
Class-unswitched memory B 11.7
Double Negative 8.93
Naïve B 65.7
Transitional B 0.14
Mature B 93.9
Plasmablasts 5.24
Class-switched memory B 40.7
Class-unswitched memory B 18.8
Double Negative 7.02
Naïve B 33.5
d
V(D)J rearrangement
Healthy
VEXAS
IGHJ1 IGHJ2 IGHJ3 IGHJ4 IGHJ5 IGHJ6 IGKJ1 IGKJ2 IGKJ3 IGKJ4 IGKJ5 IGLJ1 IGLJ2 IGLJ3 IGLJ7
IGHV1 IGHV2 IGHV3 IGHV4 IGHV5 IGHV6 IGHV7 IGKV1 IGKV2 IGKV3 IGKV4 IGKV5 IGKV6 IGLV1 IGLV10 IGLV2 IGLV3 IGLV4 IGLV5 IGLV6 IGLV7 IGLV8 IGLV9
Frequency
0.02
0.06
0.10
e
SHM frequency in B cell subclusters
SHM frequency
High (> 3%)
Low (0 - 3%)
None (No SHM)
UMAP1
UMAP2
f
SHM frequency in B cell subtypes
Naïve
IgM Memory
Classical memory
Double negative
Plasmablast
Cell proportion
Healthy
VEXAS
Somatic hypermutation frequency (SHM)
High (> 3%) Low (0 - 3%) None (No SHM)
g
Naïve B
Classical memory B
IgM memory B
Double negative B
Plasmablasts
SHM frequencies
P < 1.0 x 10-10
P = 0.067
P < 1.0 x 10-10
P < 1.0 x 10-10
P = 0.0073
h
IgM-expressed memory B
P < 1.0 x 10-10

Supplementary Fig. 7: Immunological features of B cells and TCR analysis. a, Violin plot of gene expression distributions of selected canonical cell markers in six B cell subtypes. Rows and columns indicate clusters and selected marker genes. **b**, Identification of B cell subsets in peripheral blood. **c**, Representative flow plots for B cell subsets in peripheral blood from VEXAS patients (right) and healthy donors (left). **d**, V(D)J rearrangement differences between VEXAS patients and healthy donors. Colors indicate usage percentages of specific V-J gene pairs. **e**, UMAP plot embedding subclustered B cells colored by levels of somatic hypermutation (SHM) in their corresponding BCRs. Cells with both scRNA-seq data and scBCR-seq data are shown. **f**, SHM frequency distribution in each B-cell subtype according to VEXAS and healthy donors. **g**, Comparison of SHM frequency between each B cell subtype of VEXAS patients and that of healthy donors. *P* values were calculated using the two-sided unpaired Mann-Whitney *U* test. **h**, Comparison of SHM frequency between IgM-expressed memory B cells of VEXAS patients and those of healthy donors. *p* values were calculated using the two-sided unpaired Mann-Whitney *U* test.
