# Supplementary material for: In depth transcriptomic profiling defines a landscape of dysfunctional immune responses in patients with VEXAS syndrome
Source: Nat Commun. 2025 May 20;16:4690. doi: 10.1038/s41467-025-59890-0 (PMC12092610; doi:10.1038/s41467-025-59890-0)
Supplement: Supplementary file 2 — Description of Additional Supplementary Files [file 41467_2025_59890_MOESM2_ESM.docx]

**Description of Additional Supplementary Files**

Supplementary Data 1. Clinical and laboratory characteristics of patients with VEXAS

syndrome.

Supplementary Data 2. UBA1 genotyping metrics in 10x gene expression data and

GoT libraries.

Supplementary Data 3. Differentially expressed genes in monocytes from VEXAS.

Supplementary Data 4. Differentially expressed genes in UBA1-mutated NK cells.

Supplementary Data 5. Sequences of TCR groups with more than five different clone

obtained from GLIPH2 analysis.
