# Supplementary material for: In depth transcriptomic profiling defines a landscape of dysfunctional immune responses in patients with VEXAS syndrome
Source: Nat Commun. 2025 May 20;16:4690. doi: 10.1038/s41467-025-59890-0 (PMC12092610; doi:10.1038/s41467-025-59890-0)
Supplement: Supplementary file 8 — Reporting Summary [file 41467_2025_59890_MOESM8_ESM.pdf]

nature portfolio

Corresponding author(s): Zhijie Wu

Last updated by author(s): Mar 28, 2025

Reporting Summary

Nature Portfolio wishes to improve the reproducibility of the work that we publish. This form provides structure for consistency and transparency in reporting. For further information on Nature Portfolio policies, see our Editorial Policies and the Editorial Policy Checklist.

Statistics

For all statistical analyses, confirm that the following items are present in the figure legend, table legend, main text, or Methods section.

n/a	Confirmed	
☐	☒	The exact sample size (n) for each experimental group/condition, given as a discrete number and unit of measurement
☐	☒	A statement on whether measurements were taken from distinct samples or whether the same sample was measured repeatedly
☐	☒	The statistical test(s) used AND whether they are one- or two-sided<br>*Only common tests should be described solely by name; describe more complex techniques in the Methods section.*
☒	☐	A description of all covariates tested
☐	☒	A description of any assumptions or corrections, such as tests of normality and adjustment for multiple comparisons
☐	☒	A full description of the statistical parameters including central tendency (e.g. means) or other basic estimates (e.g. regression coefficient) AND variation (e.g. standard deviation) or associated estimates of uncertainty (e.g. confidence intervals)
☐	☒	For null hypothesis testing, the test statistic (e.g. F, t, r) with confidence intervals, effect sizes, degrees of freedom and P value noted<br>*Give P values as exact values whenever suitable.*
☒	☐	For Bayesian analysis, information on the choice of priors and Markov chain Monte Carlo settings
☒	☐	For hierarchical and complex designs, identification of the appropriate level for tests and full reporting of outcomes
☒	☐	Estimates of effect sizes (e.g. Cohen's d, Pearson's r), indicating how they were calculated

Our web collection on statistics for biologists contains articles on many of the points above.

Software and code

Policy information about availability of computer code

Data collection	Single cell RNA sequencing (scRNA-seq) data was collected using an Illumina NovaSeq. Flow cytometry data was collected by BD Fortessa. Olink data was collected by Olink Signature Q100 instrument. Luminex sample data was collected by LUMINEX-200.
Data analysis	Flow cytometry data was analyzed by FlowJo (10.9.0) software. Single cell RNA sequencing data was analyzed by 10X CellRanger7.0.1 package, available from 10x Genomics (https://support.10xgenomics.com/single-cell-gene-expression/software/pipelines/latest/what-is-cell-ranger), the Seurat R package (4.0.4, http://satijalab.org/seurat/), AUCell package (1.4.1) and miloR (1.8.1) in Bioconductor, tCR package (2.3.2, https://imminfo.github.io/tcr/), GLIPH2 (http://50.255.35.37:8080/), cbsniffer (1.0, https://github.com/sridnona/cb_sniffer), IronThrone (2.1, https://github.com/dan-landau/IronThrone-GoT), Connectome (1.0.2, https://github.com/msraredon/Connectome), Slingshot (2.10.0, https://github.com/kstreet13/slingshot), Immcantation toolbox (4.0.0, https://immcantation.readthedocs.io/en/stable/index.html), and GSEA software (https://www.gsea-msigdb.org/gsea/index.jsp). Olink data was analyzed by Olink Analyze (3.7.0, https://github.com/Olink-Proteomics/OlinkRPackage). Luminex sample data was analyzed by Bio-Plex Manager (6.1.1, https://www.bio-rad.com/en-us/product/bio-plex-manager-software-standard-edition). The code for Genotyping of Transcriptomes is available at Github: https://github.com/shouguog/NHLBIGoT and Zenoda: https://doi.org/10.5281/zenodo.15046442 .

For manuscripts utilizing custom algorithms or software that are central to the research but not yet described in published literature, software must be made available to editors and reviewers. We strongly encourage code deposition in a community repository (e.g. GitHub). See the Nature Portfolio guidelines for submitting code & software for further information.

Data

Policy information about availability of data

All manuscripts must include a data availability statement. This statement should provide the following information, where applicable:

- Accession codes, unique identifiers, or web links for publicly available datasets
- A description of any restrictions on data availability
- For clinical datasets or third party data, please ensure that the statement adheres to our policy

The raw and analyzed sequencing data in this study can be found at NCBI's Gene Expression Omnibus (series accession code GSE249131) and Sequence Read Archive (accession code PRJNA1047528).

Field-specific reporting

Please select the one below that is the best fit for your research. If you are not sure, read the appropriate sections before making your selection.

☒ Life sciences ☐ Behavioural & social sciences ☐ Ecological, evolutionary & environmental sciences

For a reference copy of the document with all sections, see nature.com/documents/nr-reporting-summary-flat.pdf

Life sciences study design

All studies must disclose on these points even when the disclosure is negative.

Sample size	Sample size for the current experimental study was limited to original clinical trial. No sample size calculation was performed; sample size was determined arbitrarily based on availability of clinical samples. Nine patients with VEXAS were recruited for scRNA-seq experiment. Forty seven patients were additionally recruited for validation experiments. Blood samples were collected from subjects after written informed consent under clinical protocol.
Data exclusions	Sequencing data were processed and filtered using well-established pipeline, and only single cells passing quality control were retained for further analysis.
Replication	Biological replication dose not apply to human samples, instead, we included multiple individuals in each group for comparison. No technical replication was performed due to cost of experiments.
Randomization	The original clinical protocol (NCT05012111) was designed as an exploratory natural history protocol that enrolls patients who have been diagnosed with acquired or inherited bone marrow failure. There is a single arm and randomization is not applicable.
Blinding	The original clinical protocol (NCT05012111) was designed as an exploratory natural history protocol that enrolls patients who have been diagnosed with acquired or inherited bone marrow failure. There is a single arm and blinding is not applicable.

Reporting for specific materials, systems and methods

We require information from authors about some types of materials, experimental systems and methods used in many studies. Here, indicate whether each material, system or method listed is relevant to your study. If you are not sure if a list item applies to your research, read the appropriate section before selecting a response.

Materials & experimental systems

n/a	Involved in the study	
☐	☒	Antibodies
☒	☐	Eukaryotic cell lines
☒	☐	Palaeontology and archaeology
☒	☐	Animals and other organisms
☐	☒	Human research participants
☐	☒	Clinical data
☒	☐	Dual use research of concern

Methods

n/a	Involved in the study	
☒	☐	ChIP-seq
☐	☒	Flow cytometry
☒	☐	MRI-based neuroimaging

Antibodies

Antibodies used	We described antibodies used in the Methods section.<br>1. Anti-human CD3 (Clone SK7) APC/Cyanine7, BioLegend, Catalog#344818, RRID: AB_10645474.<br>2. Anti-human CD14 (Clone M5E2) APC/Cyanine7, BioLegend, Catalog#301820, RRID: AB_ 493695.<br>3. Anti-human CD56 (NCAM) (Clone 5.1H11) PE/Cyanine7, BioLegend, Cat# 362510, RRID: AB_2563927.<br>4. Anti-human CD16 (Clone HI10A) APC, BioLegend, Cat# 360706, RRID: AB_2562751.

5. Anti-FcεRI Antibody, γ subunit FITC, Millipore Sigma, Cat#FCBS400F
6. Anti-human CD57 (Clone HQA17A04) BV510, BioLegend, Cat# 393313, RRID: AB_2750342.
7. Mouse anti-human CD19 (Clone SJ25C1) APC/Cyanine7, BD Biosciences, Cat# 557791, RRID: AB_396873.
8. Anti-human CD20 (Clone HI100) FITC, BioLegned, Cat# 302304, RRID: AB_314252.
9. Anti-human IgD (Clone IA6-2) PE, BioLegend, Cat# 348204, RRID: AB_10553900.
10. Anti-human CD27 (Clone O323) BV785, BioLegend, Cat# 302832, RRID: AB_2562674.
11. Anti-human CD24 (Clone ML5) BV605, BioLegned, Cat# 311124, RRID: AB_2562288.
12. Anti-human CD19 (Clone HIB19) BV421, BioLegend, Cat# 302233, RRID: AB_11142678.
13. Mouse anti-human CD38 (Clone HB7) APC, BD Biosciences, Cat# 340439, RRID: AB_400512.
14. 7-AAD Viability Staining Solution, Cat#00-6993-50.

Validation

1. https://www.biolegend.com/en-gb/products/apc-cyanine7-anti-human-cd3-antibody-6940?GroupID=BLG5900
2. https://www.biolegend.com/en-gb/products/apc-cyanine7-anti-human-cd14-antibody-3293
3. hhttps://www.biolegend.com/en-gb/product-preview/pe-cyanine7-anti-human-cd56-ncam-antibody-9959?GroupID=BLG13037
4. https://www.biolegend.com/en-gb/products/apc-anti-human-cd16-antibody-9053
5. https://www.sigmaaldrich.com/US/en/product/mm/fcabs400f
6. https://www.biolegend.com/en-gb/products/brilliant-violet-510-anti-human-cd57-recombinant-antibody-16248
7. https://www.bdbiosciences.com/en-us/products/reagents/flow-cytometry-reagents/research-reagents/single-color-antibodies-ruo/APC-Cy%E2%84%A27-Mouse-Anti-Human-CD19.557791
8. https://www.biolegend.com/en-gb/products/fitc-anti-human-cd20-antibody-558
9. https://www.biolegend.com/en-gb/products/pe-anti-human-igd-antibody-6532
10. https://www.biolegend.com/en-gb/products/brilliant-violet-785-anti-human-cd27-antibody-7970
11. https://www.biolegend.com/en-gb/products/brilliant-violet-605-anti-human-cd24-antibody-8680
12. https://www.biolegend.com/en-gb/products/brilliant-violet-421-anti-human-cd19-antibody-7144
13. https://www.bdbiosciences.com/en-us/products/reagents/flow-cytometry-reagents/clinical-discovery-research/single-color-antibodies-ruo-gmp/apc-mouse-anti-human-cd38.340439
14. https://www.thermofisher.com/order/catalog/product/00-6993-50

Human research participants

Policy information about studies involving human research participants

Population characteristics

The full cohort information is available in Supplementary Table 1 (Clinical and laboratory characteristics of patients with VEXAS syndrome).

Recruitment

All participants were enrolled in research studies that had been approved by the respective institutional review boards and provided written informed consent. To be eligible to participate in this study, an individual must meet all of the following criteria.

INCLUSION CRITERIA:
-Age >=2 years
-Diagnosis of acquired or inherited bone marrow failure or ineffective hematopoiesis
-Ability and willingness to come to the NIH CC for consultation and testing
-Ability of subject or Legally Authorized Representative (LAR) to understand the investigational nature of the protocol and their willingness to sign a written informed consent document.

EXCLUSION CRITERIA:
-Post HSCT
-MDS/AML on chemotherapy (patients with hypoplastic MDS who have received or are on erythropoietin stimulating agent (ESA), granulocyte colony-stimulating factor (GCSF), or immunosuppressive treatment will not be excluded). Subjects in Cohort 3 with TBD can be seen if they have received chemotherapy for MDS/AML.

Ethics oversight

Written informed consent were under protocol (www.clinicaltrials.gov NCT05012111) approved by the Institutional Review Boards of National Heart, Lung, and Blood Institute, in accordance with the Declaration of Helsinki.

Note that full information on the approval of the study protocol must also be provided in the manuscript.

Clinical data

Policy information about clinical studies

All manuscripts should comply with the ICMJE guidelines for publication of clinical research and a completed CONSORT checklist must be included with all submissions.

Clinical trial registration

www.clinicaltrials.gov NCT05012111

Study protocol

www.clinicaltrials.gov NCT05012111

Data collection

All patients underwent comprehensive review of all available clinical records, and whenever possible, prospective clinical evaluation.

Outcomes

This is a natural history study, and patients were treated with various regiments. No treatment outcome information was used to correlate with data of the current study.

Flow Cytometry

Plots

Confirm that:

☒ The axis labels state the marker and fluorochrome used (e.g. CD4-FITC).

☒ The axis scales are clearly visible. Include numbers along axes only for bottom left plot of group (a 'group' is an analysis of identical markers).

☒ All plots are contour plots with outliers or pseudocolor plots.

☒ A numerical value for number of cells or percentage (with statistics) is provided.

Methodology

Sample preparation	PB mononuclear cells (PBMCs) were isolated by Ficoll-Hypaque density gradient centrifugation using Ficall-Paque Premium mononuclear cell separation medium (#17544202, Cytiva). Briefly, PB samples diluted twofold with phosphate buffered saline (PBS) (#10010031, Thermo Fisher Scientific) were layered on top of 1 volume Ficall-Paque medium in a 50-ml Falcon tube and centrifuged at 1,140g for 20 min at room temperature with brake off. Isolated PBMCs were treated with ACK lysing buffer (#118-156-101, Quality Biological) for lysis of red blood cells, washed with PBS. PBMCs were cryopreserved in 10% DMSO (#D2650-100ML, Thermo Fisher Scientific) in heat-inactivated FBS in liquid nitrogen until use. Cryopreserved PBMCs (5–10 × 10e6 cells) were thawed and stained with the fluorochrome-conjugated monoclonal antibodies (mAbs) listed above.
Instrument	Becton Dickinson LSR Fortessa
Software	Data were analyzed using FlowJo software (Tree Star Inc.)
Cell population abundance	According to each sample.
Gating strategy	NK cell populations were identified as CD56+Lin-(CD3&CD14&CD19). CD56bright NK cells were identified as CD16-CD56bright, CD56dim NK cells as CD16+CD56dim. Adaptive-like NK cells were identified as CD56+CD3-CD14-CD19-/CD16+CD56dim/CD57+FcεR1γ-. Mature B cells were identified as CD24±CD38+, transitional B cells as CD24+CD38+, and plasmablasts as CD24-CD38+. Mature B cells were further divided into four subtypes based on their CD24 and IgD expression: class-switched memory B (CD27+IgD-), class-unswitched memory B (CD27+IgD+), naïve B (CD27-IgD+), and double negative B (CD27-IgD-).

☒ Tick this box to confirm that a figure exemplifying the gating strategy is provided in the Supplementary Information.
